# Increasing engagement with, and effectiveness of, an online CBT-based stress management intervention for employees through the use of an online facilitated bulletin board: study protocol for a pilot randomised controlled trial

**DOI:** 10.1186/s13063-016-1733-2

**Published:** 2016-12-15

**Authors:** Stephany Carolan, Peter R. Harris, Kathryn Greenwood, Kate Cavanagh

**Affiliations:** Research and Development Department, Sussex Partnership NHS Foundation Trust and School of Psychology, University of Sussex, Falmer, BN1 9QH UK

**Keywords:** Online, Internet, CBT, Stress, Work

## Abstract

**Background:**

The evidence for the benefits of online cognitive behaviour therapy (CBT)-based programmes delivered in a clinical context is clear, but this evidence does not translate to online CBT-based stress management programmes delivered within a workplace context. One of the challenges to the delivery of online interventions is programme engagement; this challenge is even more acute for interventions delivered in real-world settings such as the workplace. The purpose of this pilot study is to explore the effect of an online facilitated discussion group on engagement, and to estimate the potential effectiveness of an online CBT-based stress management programme.

**Methods:**

This study is a three-arm randomised controlled trial (RCT) comparing a minimally guided, online, CBT-based stress management intervention delivered with and without an online facilitated bulletin board, and a wait list control group. Up to 90 employees from six UK-based organisations will be recruited to the study. Inclusion criteria will include age 18 years or over, elevated levels of stress (as measured on the PSS-10 scale), access to a computer or a tablet and the Internet. The primary outcome measure will be engagement, as defined by the number of logins to the site; secondary outcome measures will include further measures of engagement (the number of pages visited, the number of modules completed and self-report engagement) and measures of effectiveness (psychological distress and subjective wellbeing). Possible moderators will include measures of intervention quality (satisfaction, acceptability, credibility, system usability), time pressure, goal conflict, levels of distress at baseline and job autonomy. Measures will be taken at baseline, 2 weeks (credibility and expectancy measures only), 8 weeks (completion of intervention) and 16 weeks (follow-up). Primary analysis will be conducted on intention-to-treat principles.

**Discussion:**

To our knowledge this is the first study to explore the effect of an online discussion group on the engagement and effectiveness of an online CBT-based stress management intervention. This study could provide a solution to the growing problem of poor employee psychological health and begin to address the challenge of increasing engagement with Internet-delivered health interventions.

**Trial registration:**

ClinicalTrials.gov Identifier: NCT02729987. Registered on 18 Mar 2016.

**Electronic supplementary material:**

The online version of this article (doi:10.1186/s13063-016-1733-2) contains supplementary material, which is available to authorized users.

## Background

One in six adults in England meet the diagnostic criteria for at least one common mental health disorder (CMHD), but only 24% of them are receiving any form of treatment [1]. Psychological ill health is the leading cause of sickness absence in the UK accounting for 70 million sick days in 2013 and costing the economy £70–£100 billion per year [2]. Reducing the prevalence of CMHDs is a major public health challenge [1]. One approach to addressing this challenge is to utilise the Internet as a means of delivering evidence-based psychological treatments.

In 2013, 73% of adults in Great Britain used the Internet every day, with 43% using it to seek health information [3]. The Internet has become a natural means for delivering health care information [4], treatment, and prevention programmes [5]. In the UK, computerised cognitive behaviour therapy (CBT) (cCBT) is endorsed by the National Institute for Health and Care Excellence [6] for the treatment of persistent subthreshold depressive symptoms or mild to moderate depression. NICE have also identified cCBT as a promising low-intensity intervention for generalised anxiety disorder [7].

A large number of meta-analyses have found evidence for the delivery of online CBT-based programmes delivered in clinical or community settings for individuals with depression and anxiety [8–12], but the evidence for online psychological interventions delivered in workplace settings is less convincing [13–15].

Researchers have argued that adherence (completing the intervention to the extent that the developers intended it to be used; [16]), engagement (the extent, both in terms of time and frequency, that participants visit the website) and attrition (participants in a study who do not fulfill the research protocol; [16]) all pose challenges to the evaluation and delivery of Internet interventions [17–19]. For Internet interventions delivered in real-world settings (as opposed to clinical research settings), these challenges can be even more acute [20, 21] with as few as 1% of registered users completing all sessions of a freely available online CBT programme for people with panic disorder and agoraphobia [22].

Evidence suggests that increasing guidance from a therapist can lead to greater adherence to online interventions, and result in improved outcomes [8, 12, 16, 23–26]. A facilitated discussion group delivered in the form of a bulletin board could provide a cost-effective and time-efficient means for increasing guidance from a therapist. Although more evidence is needed to support this hypothesis, there is some evidence of improved adherence to bulletin board support: Titov et al. [27], compared guided and nonguided Internet-based CBT for social phobia. The guided condition had access to a facilitated bulletin board and email contact from a therapist. The unguided condition had access to a nonfacilitated bulletin board. The study found that adherence rates for the supported condition were higher than for the unsupported condition (77% and 33% respectively). What was unclear from the study was the extent to which it was the facilitated bulletin board or the email support that successfully provided the additional therapist guidance.

A number of other studies [28–30] have also included discussion groups delivered in the form of bulletin boards as part of an online intervention but have failed to include the groups as a unique research variable so have been unable to identify the impact of the group on the effectiveness of the intervention.

In this study we will examine the effect of an online facilitated bulletin board on engagement with an online CBT-based stress management programme (WorkGuru) and explore whether effectiveness is mediated by engagement. We hypothesise that the bulletin board group will have better engagement outcomes than the minimal support group (MSG), and that these outcomes will result in decreased levels of psychological distress and increased levels of subjective wellbeing at work. Furthermore, we expect to identify moderating factors that influence levels of engagement and effectiveness that are either linked to the quality of the intervention (satisfaction, acceptability, credibility, system usability), time pressure, goal conflict, level of distress at baseline, or job autonomy.

This study is being conducted as a pilot phase of a substantive trial; this will give greater confidence in predicting effect size, refining the optimum engagement of the intervention (adherence) and understanding the accuracy and effectiveness of engagement measures. It will also give a greater understanding of the challenges of conducting this research in a workplace setting.

### Aim of the study

The aim of this pilot study is to inform a definitive randomised controlled superiority trial. The objectives are:

1. To assess recruitment rate, level of study attrition and the robustness of engagement measures
2. To provide an effect size prediction
3. To get a better understanding of the extent to which participants are engaging with the modules and the bulletin board so that threshold levels of adherence can be refined
4. To identify the challenges of conducting research and delivering an online intervention in the workplace

## Methods

### Study design

**Fig. 1:**
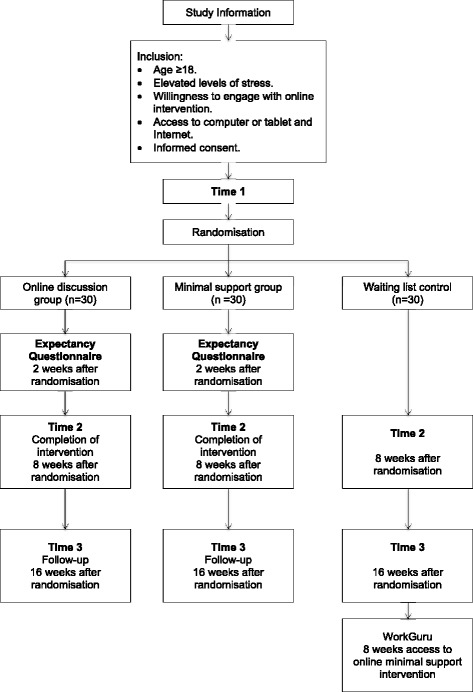
Study flow chart

A three-arm randomised controlled trial (RCT) will be conducted to compare engagement and effectiveness of a minimally guided, online, CBT-based stress management intervention (WorkGuru) delivered with and without an online facilitated bulletin board. Both active conditions will be compared with a wait list control group (WLC). All participants will have unrestricted access to Care as Usual (CAU), such as counselling and medication, which will be monitored to control for potential confounding effects. The trial will be conducted and reported in line with Consolidated Standards of Reporting Trials (CONSORT) 2010 guidelines [31]. A completed Standard Protocol Items: Recommendations for Interventional Trials (SPIRIT) 2013 Checklist (Additional file 1) and chart (see Table 1) have been completed and submitted for publication. Online assessments will be conducted before randomisation, at 2 weeks (credibility/expectancy measure only), on completion of treatment (8 weeks) and at 16-week follow-up (see Fig. 1).

**Table 1:** Standard Protocol Items: Recommendations for Interventional Trials (SPIRIT) schedule of enrolment, interventions and assessment

	Study period (weeks)								
Time point	3/16	4/16	5/16	6/16	7/16	8/16	9/16	10/16	11/16
Enrolment									
Recruitment	X	X	X	X					
Eligibility screen	X	X	X	X					
Informed consent	X	X	X	X					
Allocation	X	X	X	X					
Interventions:									
Discussion group	X	X	X	X	X	X			
Minimal support group (MSG)	X	X	X	X	X	X			
Wait list control group (access to MSG)					X	X	X	X	X
Assessments									
T1	X	X	X	X					
Credibility/expectancy	X	X	X	X	X				
T2			X	X	X				
T3					X	X	X		
Study completion									X

### Recruitment and randomisation

Six UK-based organisations will be approached to participate in this study. A sample size of 90 study participants will be recruited from the participating organisations. The sample size of 30 participants per arm is based on the optimum number of discussion group participants identified by WorkGuru, and is equal to the medium per arm sample size identified in an audit of sample sizes for pilot and feasibility studies [32]. Participants will be recruited through advertisements distributed via email, the organisations’ intranets and in-house magazines. All marketing information will include an email address inviting people who are interested in participating in the study to access information made available online or by emailing the first named author (SC). An information leaflet and a link to the online screening questionnaire, the short-form version of the Perceived Stress Scale (PSS-10; [33]) will be made available to all people who express an interest in the study. People who meet the inclusion criteria will automatically be sent a link to the online baseline questionnaire. The online questionnaires will be designed and distributed using Qualtrics survey software. Participants who complete the baseline questionnaire will be randomised. The first author will create an allocation schedule using a computer-generated randomisation sequence (random.org). An independent researcher not otherwise involved in the research will allocate each group (A, B or C) as an active condition (with or without a facilitated bulletin board) or as the WLC. Participants will be randomly allocated on a ratio of 1:1:1 to these groups. The study researchers will be blind to the group allocation.

Randomisation is being conducted at an individual level rather than at organisation or team level. This allows us to control for group stressors such as large-scale redundancies and team deadlines. One of the risks of individual-level randomisation is contamination between the groups (i.e. participants in the WLC talking with participants in an active intervention). The extent of contamination between the study groups will be monitored.

Participants using the bulletin board will be required to use a pseudonym to maintain researcher blindness. Individual-level randomisation has been chosen to control for group stressors (i.e. organisational, department or team change).

### Inclusion and exclusion criteria

Inclusion criteria will be: age 18 years or over, employed by participating organisation, willingness to engage with an online CBT-based stress management intervention, access to a computer or tablet, access to the Internet, and a score of ≥20 on the PSS-10. No exclusion criteria have been set.

### Intervention

The online CBT-based stress management intervention WorkGuru is presented on a secure platform that participants log on to using email addresses and a self-generated password. WorkGuru is a modular intervention that is based on the psychological principles of CBT, positive psychology, mindfulness and problem solving. It has been designed to increase self-awareness, improve flexible thinking and teach active coping skills. There are 10 modules that individuals can select to complete (see Table 2 for more information). Seven of those modules comprise the core modules, which all participants will be advised to complete. The modules consist of a combination of educational reading and audio, short animations, and interactive exercises. Participants can complete a questionnaire and receive suggestions for modules that they may find useful, or they can chose the modules themselves. As well as the modules, participants can complete eight self-monitoring standardised questionnaires, for example: the Perceived Stress Scale [33], the Subjective Happiness Scale [34] and the Brief Resilience Scale [35]. Participants have the option to opt in to a weekly email (the Monday Morning Message) that will reinforce messages of positive thinking and healthy working practices. They can also set themselves email reminders to visit the site. To encourage engagement, the coach will contact each participant through the site three times during the course of the 8-week programme (when an account is created, at 2 weeks and at 6 weeks). Participants can chose to share their work with the coach, and to contact the coach at anytime through the site to ask for feedback or additional help or advice. The coach will respond within 24 h. The coach has a postgraduate-level qualification in executive coaching.

**Table 2:** WorkGuru modules

	Module title	Module content	Suggested completion time
1.	Learn about me and my values^a^	Two questionnaires designed to help identify and prioritise work and life values. Exercise to prioritise those values	70 min
2.	Identify the things that cause me stress^a^	Psychoeducational information on stress, a Stress Diary and an exercise designed to help individuals analyse their stress diaries looking for patterns to their stress trigger, and the helpful and not so helpful ways they respond to stress	90 min (plus Stress Diary)
3.	Recognise the early warning signs of stress^a^	Psychoeducational information on stress and an exercise to help identify physical, psychological and behavioural symptoms of stress	20 min
4.	Learn about how I think^a^	Brief CBT including cognitive restructuring, automatic thoughts and unhelpful thinking styles	30 min (plus Thought Diary)
5.	Resilience: learning to bounce back^a^	Psychoeducational material on positive psychology and nine ‘happiness’ exercises focussing on increasing positive thinking	40 min (plus exercises to be completed over a number of weeks)
6.	Manage my stress^a^	Identifying and reducing the demands that are being made, and increasing the capacity to cope with those demands.	60 min
7.	Working smarter not harder^a^	Exercise and psychoeducational material on prioritising, focussing energy, learning to let go and time management	90 min (plus an option of a diary)
8.	Think about where I want to be in the future	Exercises to help imagine the best possible self and steps needed to get there	30 min
9.	Mindfulness	An introduction to mindfulness with guided meditations	90 min
10.	Explore creative ways to problem solve	Exercises introducing problem solving techniques	60 min

Note: ^a^Core module

Throughout the content of the WorkGuru website, users are prompted that if they are concerned about their mental health they should speak to their GP, NHS 111 or the Samaritans. Contact details are given.

### Minimal support group (MSG)

The MSG will have full access to the intervention as described above, including direct messaging support from the coach.

### Online discussion group

As well as accessing the online modules, and the direct messaging support from a coach, the online discussion group will also have access to an 8-week online facilitated discussion group delivered via a bulletin board. The discussion group will be facilitated by a coach in groups of 30. The coach will introduce one or more modules each week and encourage discussion about the topic. Participants will be required to select a user name, so that they will be anonymous in the group.

### Wait list control group (WLC)

The WLC will have access to the minimal support intervention (the online modules and direct messaging support from a coach) after 16 weeks.

### Measures

#### Screening measure

A score of ≥20 on the PSS-10 has been identified as inclusion criteria for this study. The cut-off of 20 represents 1 standard deviation (6.35) above the mean (13.02) in a large (*n* = 2387) US general population sample [36]. PSS-10 was chosen because it was felt that stress was something that employees could readily relate to (as opposed to anxiety or depression), and it is a widely used and validated scale that has been designed to measure the extent to which individuals perceive aspects of their life in that last month as being uncontrollable, unpredictable and overloading. In a review of the scale Eun-Hyun [37] reported a Cronbach’s alpha and a test-retest reliability of > .70. The author concluded that the psychometric properties of PSS-10 are superior to those of PSS-14, and recommend that PSS-10 should be used both in practice and research.

#### Primary and secondary outcome measures

The primary outcome measure of the study is engagement (the number of logins to the site); the secondary outcome measures include further engagement measures (the number of pages visited, the number of modules completed, and self-report engagement) and measures of effectiveness (the English language short-form version of the Depression, Anxiety, Stress Scale (DASS-21; [38]) and the wellbeing at work indicator (IWP Multi-affect Indicator; [39])). The DASS-21 has been designed to measure the negative emotional states of depression, anxiety and stress. In a review of the scale Henry and Crawford [40] reported a Cronbach’s alpha of .93. The scale was described as moderately sensitive to change in a clinical sample of depressed subjects [41]. The IWP Multi-affect Indicator is a 16-item measure of subjective wellbeing at work. Alpha coefficients for this scale range from .75 to .90 [42].

#### Other measures

Client Satisfaction Questionnaire [43]. The CSQ is an eight-item questionnaire that has been developed to assess general satisfaction with services. It has a high degree of internal consistency (*α* = 0.93; [43]).

A six-item questionnaire developed to rate the acceptability of cCBT was adapted from Schneider et al. [44]. Participants were asked to rate the following statements on a five-point scale where 5 is ‘strongly agree’:

1. I can use the computer at my own pace
2. Using a computer is anonymous. I don’t need to tell people about my problems
3. It is convenient for me to access help via the Internet and not to have to go to a health centre or clinic
4. I can access help at a time to suit me
5. The computer will not criticise me
6. Accessing support online is as acceptable as visiting a counsellor or other mental health professional

The credibility/expectancy questionnaire was developed by Devilly and Borkovec [45] to measure treatment expectancy and rationale credibility for the use in clinical outcome studies. An adaptation of the wording was made to replace ‘therapy’ with ‘programme’ and ‘trauma symptoms’ with ‘stress symptoms’. The authors reported high internal consistency (*α* > 0.84) and good test-retest reliability (*α* = 0.82 for expectancy and *α* = 0.75 for credibility).

The Online Support Group Questionnaire [46] was used to assess participants’ experience of the group. The authors report Cronbach alphas of the Support, Relevance and Comfort subscales as *α* = 0.84, *α* = 0.77 and *α* = 0.82, respectively.

The System Usability Scale [47] is a 10-item questionnaire which measures a subjective rating of a product’s usability. The test has demonstrated good reliability (*α* = 0.91; [48]).

The time perception measure [49] is a five-item questionnaire that has a good level of reliability (*α* = 0.89). Participants are asked to indicate on a five-point scale (originally presented by Etkin et al. as a seven-point scale) where 5 is ‘strongly agree’, the extent to which they agree with the following statements:

1. I have a sense that time is expanded
2. I have a sense that time is boundless
3. I have a sense that time is constricted
4. I always feel as if I am in a rush/hurry
5. I always feel as if I do not have enough time

The Goal Conflict Index (developed for this research) is a three-item questionnaire that asks participants to indicate on a five-point scale where 5 is ‘strongly agree’ the extent to which they agree with these statements:

1. I often feel torn between my work and my home life
2. I often have a number of competing duties that pull on my time
3. It is often difficult to prioritise between the different goals in my life

Job autonomy is measured using the nine-item autonomy subscale of the more comprehensive 77-item Work Design Questionnaire [50]. The authors report good internal consistency for the autonomy subscale (*α* > 0.85).

To test whether the programme targets a pressing concern, we have included the question: ‘On a scale of 1–10 how important is it to you to reduce your level of workplace stress?’

As recommended by Rozental et al. [51], we have included a question to identify any possible negative effects of the intervention. Deterioration between pre and post treatment will be reported, and the self-report question: ‘What, if any, positive or negative effects caused by the programme/being in the control group did you experience?’ will be asked. Existing psychological illness will be monitored with the question: ‘Have you received a diagnosis of mental illness from your GP or a health care professional?’ Contamination between the groups will be monitored with the question: ‘During the course of this study to what extent have you discussed the research with colleagues who were allocated a different research group? (For example, if you are in the control group have you spoken with colleagues who are using the online programme?)’.

CAU (including medication for depression or anxiety) will be monitored using the Client Service Receipt Inventory ([52]; adapted for this study). The CSRI was developed to be easily adaptable to the context of the research in which it is being used [53].

Previous experience of stress management training will be monitored with the question: ‘Have you previously received stress management training including training on relaxation techniques and time management?’ To assess levels of sickness absence, participants will be asked if they had taken time off work for a stress-related complaint in the previous 8 weeks at time points 1, 2 and 3.

Demographics will include: age, gender, fluency of written and spoken English, country of birth (UK, non-UK), relationship status, work role, number of working hours (low, middle, high), organisation, education level, income bracket and familiarity with online environment. The full list of measures is depicted in Table 3.

**Table 3:** Measures

Measure (number of items)	T1	T2	T3
Primary measure: engagement			
Number of logins to the website		(X)	
Secondary measures: engagement			
Number of modules completed		(X)	
Number of pages visited		(X)	
Self-report engagement		(X)	
Secondary measures: effectiveness			
DASS-21 (21)	X	X	X
IWP Multi-affect Indicator (16)	X	X	X
Other measures			
Number of visits to discussion group		X^a^	
Number of contributions to discussion group		X^a^	
Existing psychological condition	X	X	X
Messages sent to and from coach		(X)	
Demographics	X		
Care as Usual (2)	X	X	X
Time Perception Index (5)	X	X	X
Goal conflicts (3)	X	X	X
Acceptability (6)	X	X	X
Credibility/expectancy (6)		2 weeks	
Level of importance (1)	X		
Negative effects of treatment (1)		X	X
Job autonomy (9)	X		
System Usability Scale (10)		(X)	
Client Satisfaction Questionnaire (8)		(X)	
Online Support Group Questionnaire (9)		X^a^	
Experience of stress management training (1)	X		
Sickness absence for stress-related complaint (1)	X	X	X
Contamination question (1)		X	X

T1 = baseline, T2 = 8 weeks (completion of intervention), T3 = 16 weeks (follow-up)

Groups: X = all three groups, (X) = MSG and discussion group, X^a^ = discussion group only

### Statistical analyses

Analysis of the primary outcome measure (number of logins to the site) will be conducted on an intention-to-treat basis (participants’ data is analysed in the group that they are randomised to, regardless of treatment they receive or the extent to which they engage with the intervention). Analysis of the secondary measures (psychological distress and subjective wellbeing) will also be conducted on an intention-to-treat basis. Missing data will be imputed using the Last Observation Carried Forward method. For the engagement measures where no previous data is available, missing data will be imputed using the group mean. To check the robustness of the primary findings, sensitivity analysis including a per-protocol analysis will be performed. Per-protocol is defined as three or more logins to the WorkGuru site. This baseline has been identified from average login data from the site and reflects login data from other studies (for a summary of login data for online health promotion interventions see [23]).

Primary and secondary hypotheses will be explored using predominantly descriptive statistics. The means and/or medians (as appropriate) will be reported with standard deviations. Ninety-five percent confidence intervals will be calculated. Standardised effect sizes will be calculated using Cohen’s *d* and, where appropriate, odds ratios will be reported. Exploratory inferential analysis will be performed using *t* tests, analyses of variance (ANOVAs) and correlations as appropriate. In recognition that this is a pilot study caution will be taken in interpreting and reporting these results.

Baseline differences between groups will be explored using the chi-square test and ANOVA (as appropriate) and, where possible, we will compare demographics of trial participants with the workforce of each organisation to see if trial participants are representative of the workforce.

## Discussion

Workplace psychological ill-health is a growing problem for both employers and their employees, but while there is clear and convincing evidence for the efficacy of delivering CBT-based online interventions within clinical settings, that evidence has not translated to CBT-based online stress management interventions delivered within workplace settings. One explanation for this failure might be the additional challenge of achieving engagement and adherence to an online intervention that is delivered within a dynamic and busy working environment. This is also a challenge to conducting this real-world research: to what extent will a workplace setting impact on study recruitment and attrition? There is a danger that potential participants may be reluctant to engage with a stress management programme delivered via their workplace for fear of demonstrating vulnerability. We aim to counteract this by maintaining confidentiality between employee and employer. Employing organisations will not be informed of which employees are participating in the research. It may also be possible that the people that the intervention is aimed at (individuals experiencing stress) may feel so time-pressured that they are not willing to engage with the study. To counteract this while maintaining confidentiality, we will ask employers to provide a supporting statement suggesting that all employees participating in the research are given 1 h a week over the 8-week period to compete the programme. One of the aims of this pilot study is to gain a greater understanding of the ways to overcome the challenge of enabling employees to access online psychological interventions in the workplace, and to understand more about the challenges of conducting this research within a workplace setting.

Another challenge to this study is making an accurate prediction of effect size. A study by Hilvert-Bruce et al. [24] compared adherence to an online CBT programme before and after changes had been made to the way that the intervention was delivered (adding choice of course and timing, and a requirement to pay a fee). Adherence increased after the changes had been made from 37.9% to 60%, an increase of 58%. The average number of lessons completed before the changes per user was 3.72 (*SD* = 2) and 4.63 (*SD* = 1.7) after the changes. This was a significant difference *t*(1106) = 8.8, *p* < .001. The Cohen’s *d* effect size was *d* = 0.53. However, participants were recruited for the study via prescription from their GP or mental health professional. A stress management intervention, such as the one used in the present study which recruits participants with elevated (but not necessarily clinically significant) stress levels, is likely to report a smaller effect size than an intervention delivered within a clinical setting [54], which makes it difficult to calculate the predicted effect size. This pilot study, while underpowered so unable to allow us to draw definitive conclusions, will provide the parameters to inform the methods of a definitive trial.

The design of the stress management intervention (WorkGuru) is based on clear theoretical psychological principles, the efficacy for which has been proven in other studies for both face to face delivery (for example: [55, 56]), and for online delivery (for example: [57, 58]). However, the efficacy of the specific online intervention (WorkGuru) has not been established. For this reason a WLC condition has been included which will help to identify the effect of the treatment compared to no treatment. Comparing two active conditions: MSG and Discussion Group, enables the impact of the discussion group on engagement to be isolated.

Other studies have included online discussion groups as part of an online intervention (for example: [27, 29, 30]), but failed to either report usage data, differentiate the usage of the groups across the treatment groups, or analyse the impact of the discussion groups on the study outcomes. These studies are failing to include the group as a unique research variable but instead include it as one component of an intervention. This pilot study will address this failure by including the discussion group as the main research variable.

The study by Hilvert-Bruce et al. [24] found that noncompleters still benefited from the intervention but that greater adherence resulted in greater benefit. Adherence to WorkGuru has been established at three logins to the site. This baseline has been established from current WorkGuru usage and in reference to login data from studies on other health promotion sites (see [23]). This pilot study will give a greater understanding of the extent to which participants are engaging with the intervention and will enable threshold levels of adherence to be refined.

One of the stated benefits of Internet-based or eHealth (the use of information and communication technology for health) interventions is that utilisation or dose can be objectively measured [59]. The most common objective exposure measure used in studies is login rates [23], other measures include number of pages visited, length of visit and sessions or modules completed. But to what extent do these measures accurately record engagement? Computer-based utilisation measures can register whether someone visits a page but not if they have meaningfully engaged with the material. Participants’ perception of their engagement may differ from the objective utilisation measure but it is not clear to what extent that is important. Is our perception of engagement or usage a better indicator of intervention exposure than an objective utilisation measure? This pilot study will help us to get a greater understanding of designing and interpreting utilisation measures, and a greater understanding of how that relates to outcome.

This study has two active groups (the MSG and the Discussion Group). While it is not possible to blind participants to the type of intervention that they receive, which could result in a bias in the self-reported measures, the inclusion of two active groups may limit this bias. A limitation of this study is that while it focuses on engagement the quantitative nature of the study does not allow exploration of why study participants may or may not engage with the intervention. A follow-up study using qualitative methodology is being planned to address this by gaining a greater understanding of the experiences of participants who failed to engage with the study, as well as of participants who did engage.

One of the strengths of this study is that it is examining engagement and adherence to an online CBT-based stress management intervention within a real-world context (the workplace). If we want to increase access to evidence-based psychological interventions, and address the growing problem of poor employee psychological health then we need to get a better understanding of how we increase employee engagement to online psychological interventions. This study will help us to do that.

To our knowledge this is the first study that will isolate the effect of an online facilitated discussion group on adherence, engagement and effectiveness of an online CBT-based stress management intervention. This study could help to close the gap between the efficacy of online CBT-based interventions demonstrated within trials conducted in clinical settings and the effectiveness of online CBT-based interventions, delivered within real-world settings.

## Trial status

This trial began in March 2016 and is due to complete recruitment in June 2016.

## Additional file

Additional file 1: SPIRIT 2013 Checklist: recommended items to address in a clinical trial protocol and related documents*. (DOC 121 kb)

## Abbreviations

ANOVA: Analysis of variance
CAU: Care as Usual
CBT: Cognitive behaviour therapy
cCBT: Computerised cognitive behaviour therapy
CMHD: Common mental health disorder
CSQ: Client Satisfaction Questionnaire
CSRI: Client Service Receipt Inventory
DASS: Depression Anxiety Stress Scales
GP: General practitioner
MSG: Minimal support group
NICE: National Institute for Health and Care Excellence
ONS: Office for National Statistics
PSS: Perceived Stress Scale
WLC: Wait list control group

## References

[1] McManus S, Meltzer H, Brugha T, Beddington P, Jenkins R. Adult psychiatric morbidity in England, 2007: results of a household study. The NHS Information Centre for Health and Social Care; 2009. http://content.digital.nhs.uk/catalogue/PUB02931/adul-psyc-morb-res-hou-sur-eng-2007-rep.pdf.

[2] Davies SC (2014). Annual Report of the Chief Medical Officer 2013, Public Mental Health Priorities: Investing in the Evidence.

[3] ONS. Internet Access – Households and Individuals 2013. Office for National Statistics. 2013. Retrieved from http://www.ons.gov.uk/ons/dcp171778_322713.pdf. Accessed 12 Dec 2016.

[4] Tustin N (2010). The role of patient satisfaction in online health information seeking. J Health Commun.

[5] Strecher V (2007). Internet methods for delivering behavioral and health-related interventions (eHealth). Annu Rev Clin Psychol.

[6] NICE Guidelines [CG 90] (2009). Depression in adults: recognition and management.

[7] NICE Guidelines [CG113] (2011). Generalised anxiety disorder and panic disorder in adults: management.

[8] Andersson G, Cuijpers P (2009). Internet-based and other computerized psychological treatments for adult depression: a meta-analysis. Cogn Behav Ther.

[9] Andrews G, Cuijpers P, Craske MG, McEvoy P, Titov N (2010). Computer therapy for the anxiety and depressive disorders is effective, acceptable and practical health care: a meta-analysis. PLoS One.

[10] Culjpers P, Marks IM, van Straten A, Cavanagh K, Gega L, Andersson G (2009). Computer‐aided psychotherapy for anxiety disorders: a meta‐analytic review. Cogn Behav Ther.

[11] Richards D, Richardson T (2012). Computer-based psychological treatments for depression: a systematic review and meta-analysis. Clin Psychol Rev.

[12] Spek V, Cuijpers P, Nyklícek I, Riper H (2007). Internet-based cognitive behaviour therapy for symptoms of depression and anxiety: a meta-analysis. Psychol Med.

[13] Geraedts AS, Kleiboer AM, Twisk J, Wiezer NM, van Mechelen W, Cuijpers P (2014). Long-term results of a web-based guided self-help intervention for employees with depressive symptoms: randomized controlled trial. J Med Internet Res.

[14] Grime PR (2004). Computerized cognitive behavioural therapy at work: a randomized controlled trial in employees with recent stress related absenteeism. Occup Med.

[15] Philips R, Schneider J, Molosankwe I, Leese M, Foroushani PS, Grime P, Thornicroft G (2014). Randomized controlled trial of computerized cognitive behavioural therapy for depressive symptoms: effectiveness and costs of a workplace intervention. Psychol Med.

[16] Kelders SM, Kok RN, Ossebaard HC, Van Gemert-Pijnen JE (2012). Persuasive system design does matter: a systematic review of adherence to web-based interventions. J Med Internet Res.

[17] Cavanagh K, Millings A (2013). (Inter)personal Computing: The role of the therapeutic relationship in E-mental health. J Contemp Psychother.

[18] Eysenbach G (2005). The law of attrition. J Med Internet Res.

[19] Kohl LF, Crutzen R, de Vries NK (2013). Online prevention aimed at lifestyle behaviors: a systematic review of reviews. J Med Internet Res.

[20] Christensen H, Griffiths KM, Korten AE, Brittliffe K, Groves C (2004). A comparison of changes in anxiety and depression symptoms of spontaneous users and trial participants of a cognitive behavior therapy website. J Med Internet Res.

[21] Gilbody S, Littlewood E, Hewitt C, Brierley G, Tharmanathan P, Barkham M (2015). Computerised cognitive behaviour therapy (cCBT) as treatment for depression in primary care (REEACT trial): large scale pragmatic randomised controlled trial. BMJ.

[22] Farvolden P, Denisoff E, Selby P, Bagby RM, Rudy L (2005). Usage and longitudinal effectiveness of a web-based self-help cognitive behavioral therapy program for panic disorder. J Med Internet Res.

[23] Brouwer W, Kroeze W, Crutzen R, de Nooijer J, de Vries NK, Brug J, Oenema A (2011). Which intervention characteristics are related to more exposure to Internet-delivered healthy lifestyle promotion interventions? A systematic review. J Med Internet Res.

[24] Hilvert-Bruce Z, Rossouw PJ, Wong N, Sunderland M, Andrews G (2012). Adherence as a determinant of effectiveness of Internet cognitive behavioural therapy for anxiety and depressive disorders. Behav Res Ther.

[25] Mohr DC, Cuijpers P, Lehman K (2011). Supportive accountability: a model for providing human support to enhance adherence to eHealth interventions. J Med Internet Res.

[26] Palmqvist B, Carlbring P, Andersson G (2007). Internet-delivered treatments with or without therapist input: does the therapist factor have implications for efficacy and cost?. Expert Rev Pharmacoecon Outcomes Res.

[27] Titov N, Andrews G, Choi I, Schwencke G, Mahoney A (2008). Shyness 3: randomized controlled trial of guided versus unguided Internet-based CBT for social phobia. Aust N Z J Psychiatry.

[28] Andersson G, Bergstrom J, Hollandare F, Carlbring P, Kaldo V, Ekselius L (2005). Internet-based self-help for depression: randomised controlled trial. Br J Psychiatry.

[29] Berger T, Caspar F, Richardson R, Kneubuhler B, Sutter D, Andersson G (2011). Internet-based treatment of social phobia: a randomized controlled trial comparing unguided with two types of guided self-help. Behav Res Ther.

[30] El Alaoui S, Ljótsson B, Hedman E, Kaldo V, Andersson E, Rück C, Lindefors N (2015). Predictors of symptomatic change and adherence in Internet-based cognitive behaviour therapy for social anxiety disorder in routine psychiatric care. PLoS One.

[31] Schulz KF, Altman DG, Moher D, for the CONSORT Group. CONSORT 2010 (2010). Statement: updated guidelines for reporting parallel group randomised trials. BMJ.

[32] Billingham SA, Whitehead AL, Julious SA (2013). An audit of sample sizes for pilot and feasibility trials being undertaken in the United Kingdom registered in the United Kingdom Clinical Research Network database. BMC Med Res Methodol.

[33] Cohen S, Kamarck T, Mermelstein R (1983). A global measure of perceived stress. J Health Soc Behav.

[34] Lyubomirsky S, Lepper H (1999). A measure of subjective happiness: preliminary reliability and construct validation. Soc Indic Res.

[35] Smith BW, Dalen J, Wiggins K, Tooley E, Christopher P, Bernard J (2008). The brief resilience scale: assessing the ability to bounce back. Int J Behav Med.

[36] Cohen S, Williamson GM, Spacapan S, Oskamp S (1988). Perceived stress in a probability sample of the United States. The social psychology of health.

[37] Eun-Hyun L (2012). Review of the psychometric evidence of the Perceived Stress Scale. Asian Nurs Res.

[38] Lovibond SH, Lovibond PF (1995). Manual for the Depression Anxiety Stress Scales.

[39] Warr P (1990). The measurement of wellbeing and other aspects of mental health. J Occup Psychol.

[40] Henry JD, Crawford JR (2005). The short-form version of the Depression Anxiety Stress Scales (DASS-21): construct validity and normative data in a large non-clinical sample. Br J Clin Psychol.

[41] Page AC, Hooke GR, Morrison DL (2007). Psychometric properties of the Depression Anxiety Stress Scales (DASS) in depressed clinical samples. Br J Clin Psychol.

[42] Warr P, Bindl UK, Parker SK, Inceoglu I (2014). Four-quadrant investigation of job-related affects and behaviours. Eur J Work Organ Psy.

[43] Larsen DL, Attkisson CC, Hargreaves WA, Nguyen TD (1979). Assessment of client/patient satisfaction: development of a general scale. Eval Program Plann.

[44] Schneider J, Bennett K, Bennett A, Grime P, Grove B, Sarrami Foroushani P, Walker P. Computerised CBT for common mental disorders: RCT of a workplace intervention, Report to British Occupational Health Research Foundation. 2012. p. 1–47.

[45] Devilly GJ, Borkovec TD (2000). Psychometric properties of the credibility/expectancy questionnaire. J Behav Ther Exp Psychiatry.

[46] Chang T, Yeh CJ, Krumboltz JD (2001). Process and outcome evaluation of an on-line support group for Asian American male college students. J Couns Psychol.

[47] Brooke J, Jordan PW, Thomas B, Weerdmeester BA, McClelland IL (1996). SUS: A ‘quick and dirty’ usability scale. Usability evaluation in industry.

[48] Bangor A, Kortum PT, Miller JT (2008). An empirical evaluation of the System Usability Scale. Int J Hum Comput Interact.

[49] Etkin J, Evangelidis I, Aaker J (2015). Pressed for time? Goal conflict shapes how time is perceived, spent, and valued. J Market Res.

[50] Morgeson FP, Humphrey SE (2006). The Work Design Questionnaire (WDQ): developing and validating a comprehensive measure for assessing job design and the nature of work. J Appl Psychol.

[51] Rozental A, Andersson G, Boettcher J, Ebert DD, Cuijpers P, Knaevelsrud C, Carlbring P (2014). Consensus statement on defining and measuring negative effects of Internet interventions. Internet Interventions.

[52] Beecham J, Knapp M, Thornicorft G, Brewin C, Wing J (1992). Costing psychiatric interventions. Measuring mental health needs.

[53] Beecham J, Knapp M, Thornicroft G, Brewin C, Wing J (1999). Costing psychiatric interventions. Measuring Mental Health Needs.

[54] Tan L, Wang M-J, Modini M, Joyce S, Mykletun A, Christensen H, Harvey SB (2014). Preventing the development of depression at work: a systematic review and meta-analysis of universal interventions in the workplace. BMC Med.

[55] Blonk R, Houtman I, Brenninkmeijer V, Lagerveld SE (2006). Return to work: a comparison of two cognitive behavioural interventions in cases of work-related psychological complaints among the self-employed. Work Stress.

[56] van der Klink J, Blonk R, Schene AH, van Dijk FJ (2001). The benefits of interventions for work-related stress. Am J Public Health.

[57] Hasson D, Anderberg UM, Theorell T, Arnetz BB (2005). Psychophysiological effects of a web-based stress management system: a prospective, randomized controlled intervention study of IT and media workers [ISRCTN54254861]. BMC Public Health.

[58] Rose RD (2014). Self-guided multimedia stress management and resilience training. J Posit Psychol.

[59] Norman GJ, Zabinski MF, Adams MA, Rosenberg DE, Yaroch AL, Atienza AA (2007). A review of eHealth interventions for physical activity and dietary behavior change. Am J Prev Med.

